# Immune Homeostasis in Epithelial Cells: Evidence and Role of Inflammasome Signaling Reviewed

**DOI:** 10.1155/2015/828264

**Published:** 2015-08-19

**Authors:** Paul M. Peeters, Emiel F. Wouters, Niki L. Reynaert

**Affiliations:** ^1^Department of Respiratory Medicine, Nutrim School for Nutrition, Toxicology and Metabolism, Maastricht University Medical Centre, 6229 HX Maastricht, Netherlands; ^2^IUF-Leibniz Research Institute for Environmental Medicine, Auf'm Hennekamp 50, 40225 Düsseldorf, Germany

## Abstract

The epithelium regulates the interaction between the noxious xenogenous, as well as the microbial environment and the immune system, not only by providing a barrier but also by expressing a number of immunoregulatory membrane receptors, and intracellular danger sensors and their downstream effectors. Amongst these are a number of inflammasome sensor subtypes, which have been initially characterized in myeloid cells and described to be activated upon assembly into multiprotein complexes by microbial and environmental triggers. This review compiles a vast amount of literature that supports a pivotal role for inflammasomes in the various epithelial barriers of the human body as essential factors maintaining immune signaling and homeostasis.

## 1. Introduction

Inflammation is an extremely complex and fascinating weapon in mammalian physiology. It is the body's immediate and carefully orchestrated response to pathogens, noxious stimuli, or physical injury. In addition, responsiveness to plasma- and cell-derived inflammatory mediators reflects a more general role for inflammation in restoring functionality of the system to basal homeostatic set points. The process, by which acute inflammation is initiated and develops via molecular and cellular pathways, is well defined [1]. In case the body does not succeed in eliminating or neutralizing this condition over time, a chronic inflammatory state arises and resets the body's reference points and will become maladaptive. This implicates elevated concentrations of cytokines and chemokines including interleukin- (IL-) 1*β*, IL-1*α*, IL-6, IL-8, IL-10, IL-18, TNF-*α*, and alarmins such as high-mobility group box 1 (HMGB1). These molecules have also been proven to be involved in the progression of chronic inflammatory disorders, infections, and fibrotic diseases as well as cancer, autoimmune, and ageing-associated disorders.

It is demonstrated that IL-1 and IL-18 driven inflammation, through inflammasome activation, is initiated by recognition of endogenous or exogenous danger signals. IL-1*β*, one of the main classic instigators of inflammation is, together with IL-18, released from the cell upon activation of the inflammasome. It has the ability to affect various biological properties and has several roles in the proinflammatory response, including activation of the endothelium and leukocytes. Back in 1984 it was predicted that IL-1 was responsible for many of the acute responses to infection and inflammation [2]. IL-18, a member of the IL-1 cytokine super family, is recognized as an important regulator of innate and acquired immune responses. Its importance is derived from its prominent biological property of inducing interferon (IFN) *γ*. IL-18 is expressed at sites of chronic inflammation, in autoimmune diseases, in a variety of cancers, and in the context of numerous infectious diseases [3]. In addition to IL1 and IL18, basic fibroblast growth factor (bFGF) and HMGB1 are unconventionally released under caspase-1 activated conditions following a number of posttranslational modifications [4]. Therefore, we included bFGF as well as HMGB1 to be reviewed among the factors that could possibly be released from the epithelial cells upon inflammasome activation. For HMGB1, its earliest functions were described as a nonhistone DNA-binding nuclear protein. It facilitates DNA transcription, replication, and repair. When secreted, it has an important danger signaling and inflammation-promoting activity. It forms highly inflammatory complexes with single stranded (ss) DNA, lipopolysaccharide (LPS), IL-1*β*, and nucleosomes and interacts with toll-like receptor (TLR) 9, TLR4, IL-1R, and TLR2 and the receptor for advanced glycation end-products (RAGE). It has also been reported that HMGB1 can signal via the well-characterized nuclear factor-*κ*B pathway to induce a release of proinflammatory cytokines, including TNF-*α* and IL-1*β*. bFGF, a mitogenic agent, is not directly proinflammatory but can potentiate the recruitment of immune cells to the site of inflammation. This growth factor supports the maintenance of undifferentiated cells and is involved in tissue repair, regeneration, and proliferation. It is a potent fibrogenic mediator that can be induced by a variety of molecules in different cell types such as by silica in lung epithelial cells [5, 6] from which it is released in an inflammasome-dependent mode-of-action and potentially linked to fibroblast proliferation [7]. Activation of inflammasome-dependent mediator release is typically a very potent reaction and by virtue of the potentially destructive proinflammatory effects of uncontrolled cytokine and alarmin release their reduction and shutdown mechanisms (e.g., IL-1 receptor antagonist (IL-1RA) signaling) should therefore be tightly regulated by innate sensors.

**Figure 1:**
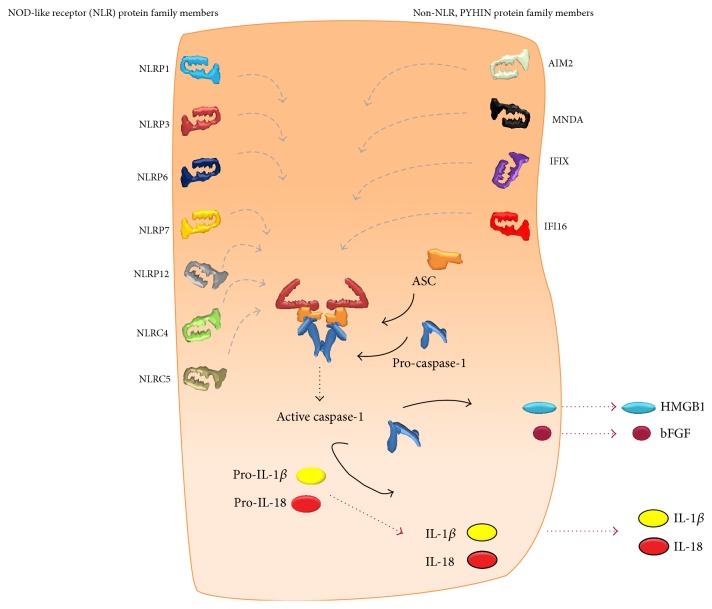
Schematic representation of all intracellular nucleotide-binding oligomerization domain receptors, in short NOD-like receptor (NLR), and pyrin and HIN200 domain-containing (PYHIN) inflammasome members, which are each able to assemble with the protease caspase-1 via the adaptor molecule apoptosis-associated speck-like protein containing a CARD (ASC) when triggered. This allows the activated enzyme to cleave and mature proinflammatory cytokines, interleukin- (IL-) 1*β*, and IL-18 as well as inducing the unconventional release of basic fibroblast growth factor (bFGF) and high-mobility group box 1 (HMGB1).

To date, a number of cytosolic receptors are successful in the recognition of conserved molecular patterns termed pathogen- or danger-associated molecular patterns (PAMPs or DAMPs), by initiating the formation of an inflammasome [8]. These include the nucleotide-binding oligomerization domain receptors, in short NOD-like receptor (NLR) protein family members NLRP1, NLRP3, NLRP6, NLRP7, NLRP12, NLRC4, and NLRC5 as well as the non-NLR pyrin and HIN200 domain-containing (PYHIN) protein family members absent in melanoma 2 (AIM2), myeloid nuclear differentiation antigen (MNDA), interferon inducible protein X (IFIX), and interferon alpha-inducible protein 16 (IFI16) which are all able to oligomerize into a functional inflammasome [9–11]. This implies the formation of a multiprotein complex consisting otherwise also of an adaptor protein, apoptosis-like speck protein containing a CARD (ASC), and IL-1*β* converting enzyme (ICE, caspase-1), the enzyme responsible for maturation of proinflammatory cytokines [12]. Upon this assembly, caspase-1 undergoes autocatalytic activation into heterotetramers, which further enables the cleavage of its substrates pro-IL-1*β* and pro-IL-18 into mature IL-1*β* and IL-18, and the unconventional release of bFGF and alarmins by the cell as is illustrated in Figure 1 [13]. In addition, caspase-1 is associated with the release of IL-1*α* [4], although these mechanisms are poorly understood. Inflammasome activation is thus linked to the most important mediators of inflammation. Moreover, their release may be accompanied by pyroptosis, an incompletely characterized proinflammatory mode of cell death [14]. Upon activation by a myriad of signaling pathways these inflammasome-dependent mediators are strongly expressed by monocytes, tissue macrophages, and dendritic cells but are also produced by B lymphocytes, natural killer (NK) cells, and epithelial cells. Activated inflammasomes and subsequently activated and released cytokines and alarmins play a key and well-controlled role in innate immunity of the lung mucosa and interstitial microenvironment. As mediators of the acute phase of inflammation they are extremely important for the immune system to react to invading pathogens. It is however equally important that inflammasomes distinguish pathogenic from nonpathogenic commensals, implying that a disturbance in normal danger signaling through the inflammasome can act as a master switch between tolerance and sensitization in many actively participating tissues [15–19].

The activation of inflammasomes in myeloid innate immune cells as well as their contribution to acute and chronic inflammatory diseases has been characterized profoundly over the past 12 years by* in vivo* and* in vitro *research [20]. Nevertheless, we are still far away from understanding how these molecules actually become activated, how they exert their function, and how they can be targeted in therapy. Aside from the specialized cells of the immune system that evolutionary developed to protect organ systems, most foreign pathogens and noxious stimuli are also encountered by epithelial cells in a barrier lining the organs that are in most proximal contact with the exterior environment [21]. These are the tissue specific mucosae of the skin, the lung, the gut, and the urogenital tract, as well as nonkeratinizing squamous epithelial cells of the oral mucosa.

The epithelium is more and more appreciated to be less passive than what was assumed before and evidence is mounting that it participates not only in receiving and relaying inflammatory signals, but in functions as an initial sensor of danger and executor of the response as well. We therefore review here the literature on the presence and functionality of inflammasomes in epithelial cells of the various organs exposed to the exterior milieu in response to reported insults. The aim of this review is to deliver better understanding of inflammatory responses of first-line barrier epithelium in multiple organs and mucosal immunity and encourage further laboratory research to dissect out the role of epithelial inflammasomes to these processes, with more effective therapies for the numerous debilitating diseases with an acute and chronic inflammatory component as the ultimate goal.

## 2. Skin

In addition to its properties as a physical barrier, the skin has many active defense mechanisms. Keratinocytes can detect microbial or nonmicrobial danger signals and elicit an immune response prior to the infiltration of myeloid cells [22]. Although the action and importance of IL-1*β*, IL-18, and HMGB1 in inflammatory skin disorders are not completely understood, dysregulation of these inflammasome-dependent molecules is an attractive concept that might play a role in many inflammatory abnormalities of the skin. In 1990 already, protein levels of immunoreactive IL-1*β* were shown to be elevated in psoriatic lesions whereas the amount released by normal keratomes or cultured keratinocytes was undetectable. The presence of IL-1*β* was suggested to be due to a novel mechanism of posttranslational processing in the epidermis [23]. This mechanism was identified in 1997 as caspase-1 dependent cleavage which could be induced in human keratinocytes in response to inflammatory and immunologic stimuli [24].

**Figure 2:**
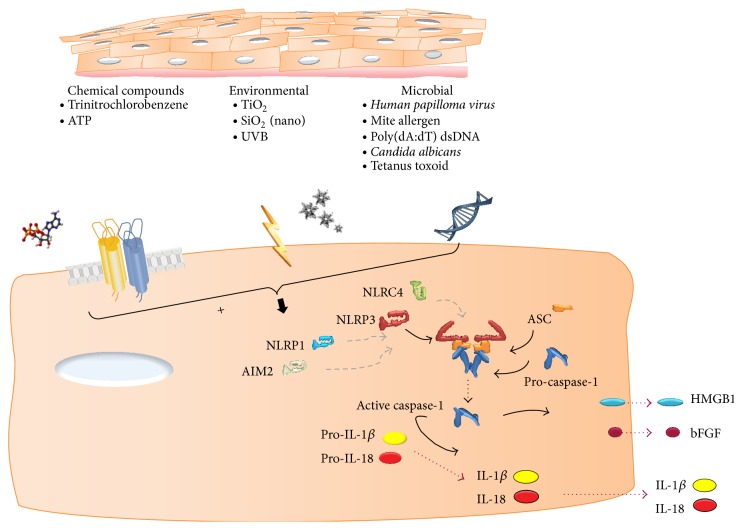
Schematic representation of stratified squamous epithelium of the skin and oral mucosa. Toxic molecules and environmental and cellular stressors as well as microbial antigens can individually activate one or more inflammasome subtypes, leading to caspase-1 activation and the release of IL-1*β*, IL-18, bFGF, and HMGB1.

Later, keratinocytes of the nondiseased skin were scarcely stained positive for NLRP1 and NLRP3 [25] and AIM2-like receptor (ALR) inflammasomes [26]. These expression patterns suggest that multiple inflammasomes are likely to play a role in the first line of defense against noxious molecules. With respect to different functional inflammasomes in the skin, human keratinocytes express AIM2 and respond to poly(dA:dT) dsDNA with IL-1*β* secretion [26]. Recently, these findings have been supported by detection of active IL-1*β* and cleaved caspase-1 in* human papillomavirus* (HPV) infected skin, suggesting inflammasome activation by viral DNA [27]. Watanabe et al. also demonstrated that the NLRP3 inflammasome is present and can be activated in keratinocytes [19] as in animal models of contact hypersensitivity this inflammasome was identified as a key regulator of innate immunity [19]. Keratinocytes are obviously also barrier cells against environmental pollutants such as TiO_2_ and SiO_2_. It was shown that these environmental particles in the nanosize could induce cleavage of caspase-1 and secretion of IL-1*β* [28]. Caspase-1 activity of stratum corneum and serum IL-18 level were also increased in patients with Netherton syndrome, a disease characterized by chronic skin inflammation [29]. In a tetanus toxoid-dependent experimental model using cocultures of monocytes and keratinocytes, others furthermore observed high levels of IL-1*β* when tetanus toxoid and keratinocytes were present, Indicating that skin epithelial cells are able to secrete caspase-1 and a source for IL-1*β* [30] (Figure 2).

In skin injury models relevant to the development of cancer, irradiation with a physiological dose of UVB induced secretion of pro-IL-1*α* and of mature and active IL-1*β* and IL-18 in a caspase-1 dependent fashion [31, 32]. Other studies of UVB overexposure in sunburned skin demonstrated activated inflammasomes [33] and UV light exposure stimulated bFGF and HMGB1 release by keratinocytes as well [34, 35]. In an allergic skin disease model, mite allergen* Dermatophagoides pteronyssinus 1* (*Der p1*, a major allergen of house dust mite), is recognized as a danger signal, activated caspase-1, and induced release of IL-1*β* and IL-18 from keratinocytes which was dependent on the cysteine protease activity. Moreover,* Der p1* stimulated assembly of the inflammasome by recruiting ASC, caspase-1, and NLRP3 to the perinuclear region [36].

The data reviewed in this section demonstrate that keratinocytes are a potent source of cytokines and alarmins upon contact with a broad spectrum of activators. It is clear that keratinocytes do not only have a passive role as target cells in the process of inflammation but also act as stimulators of the initiation and maintenance of local immune reactions.

## 3. Oral Mucosa

The oral mucosa is exposed to high density and diversity of potential microbial pathogens such as Gram-positive and Gram-negative bacteria as well as fungi and others and therefore has the important function of acting as a physical barrier and responding to microbial growth and invasion. The inflammasomes, as intracellular immune receptors, are thus likely to be important mediators of the inflammatory response in gingival epithelial cells. In a recent study depletion of NLRP3 by siRNA abrogated the ability of ATP to induce IL-1*β* secretion in infected cells [37]. ATP is sensed by purinergic receptors such as P2X, ligand-gated ion channel 4 (P2X4). Besides numerous reports on the role of P2X7 receptors in ATP-mediated inflammasome activation and mature IL-1*β* production in macrophages* in vitro*, recently in gingival epithelial cells that were stimulated with extracellular ATP, a role for P2X7 dependent-ROS production in the activation of the inflammasome was revealed (Figure 2) [38]. Its role* in vivo* has recently been questioned. Interestingly, in addition to the NLRP3 inflammasome, a different inflammasome containing NLRC4 appeared to function in the protection against infection with* Candida albicans* in the mucosal lining of the mouth and intestines rather than in immune cells [39]. No further evidence can be found on how immune homeostasis via inflammasome signaling is maintained in this environment. These studies reveal the epithelial-specific roles of the NLRP3 and NLRC4 inflammasome in innate immune response of the oral mucosa.

## 4. Gut

In organs where a variety of cell types come in intimate contact with commensals and potentially pathogenic microbes, such as the gut, the regulation and maintenance of normal intestinal mucosal barrier function is primordial for the host's survival and fitness. When cellular integrity and functioning of tight junctions between adjacent epithelial cells is disrupted barrier impairment is easily provided resulting in inflammation and the induction of tissue-repair responses. The lack of control of this inflammatory condition is suggested to aggravate in the direction of detrimental chronic inflammation in the gut. Inflammasome-dependent mediators such as IL-1*β*, IL-18, and HMGB1 have been identified as potent promoters of intestinal pathology, which suggests that targeting these mediators may represent a useful therapeutic approach in inflammatory bowel disease (IBD) [40].

Initially, observations suggested that induction of IL-1*β* mRNA in enterocytes was causally related to the subsequent inflammatory changes seen in a model of acute experimental colitis [41]. It was proposed that colon epithelial cells were programmed to provide a set of signals for the activation of the mucosal inflammatory response in the earliest phases after microbial invasion [42, 43]. Later, a few studies demonstrated that intestinal epithelial cells (IEC), continuously exposed to dietary molecules, microbial antigens, and environmental influences, played a much more active role in the host immune and inflammatory response via the secretion of a variety of cytokines limited to not only IL-1*β*, but also IL-1*α* and IL-8 [44–46]. That same year, for the first time, mouse IECs were proven to be the main producers of IL-18, formerly called interferon-gamma-inducing factor [47] under normal physiological conditions, suggesting that its constitutive expression in IECs may have an important role in the induction of mucosal immunity [48]. Two years later, IL-18 was demonstrated to be localized and increasingly expressed in intestinal mucosal cells of patients with Crohn's disease (CD) [49, 50]. Within this same period, posttranslational activation of IL-18 by caspase-1 cleavage was identified to occur in response to viral and bacterial infections [51, 52]. Specifically, a year before inflammasomes were characterized, cleavage of IL-18 in porcine intestinal mucosa by* Salmonella choleraesuis* was demonstrated, indicating that caspase-1 activation of IL-18 may be a key step in mucosal immune response to bacterial invasion [53]. Expression of IL-18 in human gastric mucosal epithelial cells was also increased by* Helicobacter pylori* infection or by lactoferrin [54, 55]. Recently, human IECs showed the ability to release IL-18 upon* Salmonella* treatment in a caspase-1 dependent fashion [56] and release HMGB1 in their culture medium upon stimulation with LPS [57] and a mixture of TNF-*α*, IL-1*β*, and IFN-*γ* [58].

**Figure 3:**
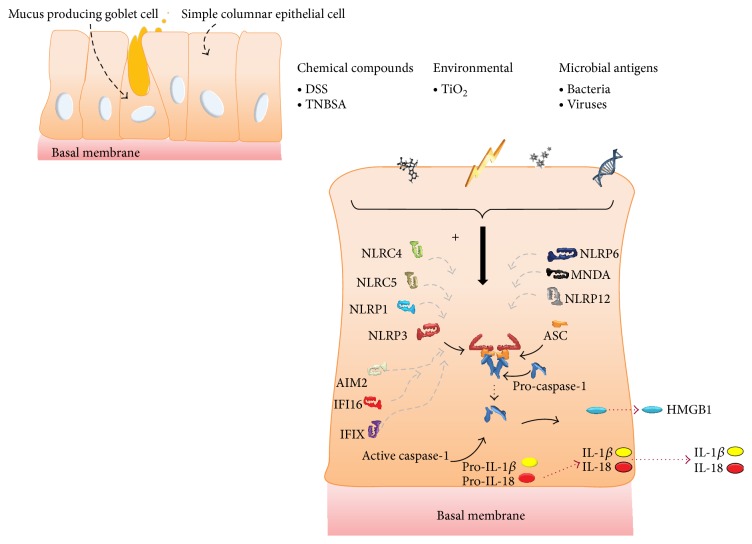
Schematic representations of simple columnar epithelial cells lining the digestive tract reflecting multiple inflammasomes that are described to be activated by different described agents AIM2, IFI16, MNDA, and PYHIN1 are subtypes of the non-NLR inflammasome family.

Microbial activity is required to be constantly monitored in the epithelial lining of the gut. It has become evident that a range of inflammasome family members within different cell types (e.g., epithelial and hematopoietic cells) accomplish different, but often complementary, functions, as watchful guardians eliciting mucosal immune responses when activated [59]. The most intensively studied inflammasomes in the gut, the NLRP1, NLRP3, NLRP6, and NLRC4 inflammasomes, have been shown to regulate a number of common intestinal mucosal infections. Importantly, different enteric infections are sensed by and linked to different inflammasome functionalities. For instance, NLRP3 and NLRC4 activation in the intestinal epithelium is essential for regulation of permeability and epithelial regeneration through sensing of commensal microbes and has been shown to protect against mucosal pathogens [60, 61]; however, excessive inflammasome activation within the lamina propria contributes to severe intestinal inflammation [62]. Moreover, whereas the NLRP6 inflammasome subtype regulated colonic microbial ecology and risk for colitis [63], it was also shown to be involved in control of epithelial self-renewal and colorectal carcinogenesis upon injury [64]. Otherwise, NLRP6 inflammasome-deficient mice have been shown to be unable to clear enteric pathogens from the mucosal surface, rendering them highly susceptible to persistent infection [65]. Additionally, recent findings suggest that both hematopoietic- and nonhematopoietic-derived NLRP12 contributed to inflammation in an experimental colitis model, but the latter dominantly contributed to tumorigenesis. Herein, NLRP12 was profiled as an important add-on in the inflammasome repertoire and new player in colonic inflammation and tumorigenesis [66]. Together, these studies reveal intensive and integrated signaling from multiple inflammasomes to regulate inflammation-induced IBD and colon cancer. In addition, unpublished data report the upregulation of most inflammasome sensor subtypes (NLRP1, NLRP3, NLRP12, NLRC4, AIM2, IFI16, MNDA, and PYHIN1) in the colonic mucosa of active IBD patients, with the double-stranded (ds) DNA responding PYHIN inflammasome subtypes (AIM2 and IFI16) showing the strongest increase. These data are accompanied with enhanced levels of IL-1*β* in primary IECs in culture following dsDNA exposure. Immunohistochemical data show, next to inflammatory cells, an epithelial presence of these inflammasome sensor subunits and some of their effector molecules (CASP1 and HMGB1) (unpublished data). Together, this indicates that a more profound focus on non-NLR signaling may be justified in IBD. The multiple activators of a broad spectrum of inflammasome subtypes implying caspase-1 activation and subsequent secretion of specific readouts in IECs are summarized in Figure 3.

Activation of intestinal inflammasomes in different lineages of cells regulates physiological reactions, and their hyperactivation or absence can lead to deleterious consequences such as inflammation or cancer progression as shown in different models [59]. For instance, following tissue damage using the IEC cytotoxic agent dextran sodium sulphate (DSS), the NLRP3 inflammasome assembles, leading to the production of IL-18, which is then released at the mucosal sites [67]. Defective NLRP3 inflammasome subtype activation was shown to protect against loss of epithelial integrity and mortality during DSS-induced experimental colitis [68], suggesting that genetic and environmental factors may activate the NLRP3 inflammasome [69]. In addition, their absence rather than their overproduction could be considered deleterious, indicating a multifaceted regulatory role of NLRP3 in intestinal inflammation. Normand et al., on the other hand, demonstrated that NLRP6-deficient mice were highly susceptible to experimental colitis [64]. Further, it was shown in humans with a leaky intestinal barrier (such as seen in IBD patients) that TiO_2_ microparticles were taken up by IEC and could activate the inflammasome and induce IL-1*β* and IL-18 secretion in the mucosa of Crohn's disease patients, representing a possible mode of aggravation of inflammation in susceptible individuals [70]. Others have shown that 2,4,6-trinitrobenzene sulfonic acid (TNBSA) was unable to induce significant colitis in IL-18 deficient mice and that administration of an IL-18 neutralizing antibody resulted in a dramatic attenuation of mucosal inflammation. The proposed function for the NLR and non-NLR inflammasomes is to regulate secretion of IL-18 that stimulates epithelial cell barrier function and regeneration, whereas, in hematopoietic cells, inflammasome activation would have a proinflammatory effect [63, 71]. This suggests that signals produced by the IECs may play an important role in inducing the early host inflammatory response to infection and raises the possibility that interventions that directly target production of inflammatory cytokines by IECs might alter the course of disease. When comparing results of studies by different groups, one should take into consideration that many of the observed effects may be explained by defective inflammasome regulation of the composition of the microflora coupled with differences in native microflora in different facilities [59].

Targeting mediator release that is associated with mucosal inflammasome activation in the gut could lead to better understanding of which pathological aspects of inflammation and subsequent increases in permeability contribute to the development of IBD.

## 5. Lung

Barrier epithelia, such as the airway epithelial cells lining the respiratory tract, fulfill multiple functions essential for tissue homeostasis. They are, because of the immense surface area that is in intimate contact with the environment, a primary target of attack by microorganisms and potentially harmful factors during every single breath. A vicious cycle of exaggerated responses to chronic stimuli or aberrant responses to rather innocent agents may result in chronic inflammation with permanent structural changes in barrier properties, including smooth muscle hyperplasia, airway remodeling, and fibrosis. The importance of engagement of pattern recognition receptors (PPRs) and their activation is demonstrated via experimental studies in knockout mice. These resulted in evidence suggesting a deleterious role for excessive production of the inflammasome-dependent proinflammatory cytokines and danger signals IL-1*β*, IL-18, and HMGB1 and the growth factor bFGF which possess multiple pathogenic properties that could be further enhanced during episodes of disease exacerbations [72–76]. Because the localization and the impact of inflammasome sensor activation in airway epithelial cells associated with pulmonary inflammation have yet to be revealed, the activation of different inflammasomes in lung epithelium in response to triggers relevant to the main chronic inflammatory diseases, asthma, COPD, pulmonary fibrosis, and pneumoconiosis, mainly in* in vitro *settings is summarized in this section.

Historically, with respect to evidence of inflammasome activation or mediator release from lung epithelium one has to go back more than a decade prior to the first characterization of the inflammasome. Immunoreactive IL-1*β* was shown to be released from bronchial epithelial cells exposed to toluene diisocyanate [77] or nitrogen dioxide [78, 79] and in tracheal biopsy material from individuals exposed to endotoxin-contaminated grain dust. A few years later, in 1996 Hastie et al. showed low but significantly higher amounts (2-fold) of IL-1*β* released from bronchial epithelial cells from allergic compared to nonallergic individuals following segmental challenge with ragweed [80]. Moreover, in experiments with exposure to another allergen,* Der p1*, cultured human airway epithelial cells were shown to release IL-1*β* [81]. In 1998, the IL-1*β* release for the first time was linked to ICE expression in alveolar epithelial cells upon* respiratory syncytial virus* (RSV) infection. Interestingly this study showed that this occurred in the absence of apoptosis [48], which could imply that epithelial cell death occurred through what is now known as pyroptosis.

Different animal models of pulmonary fibrosis have been developed to investigate the pathogenic mechanism and potential therapies for idiopathic pulmonary fibrosis (IPF). The most common is the bleomycin model in rodents (mouse, rat, and hamster) [82]. In 2001, caspase-1 mRNA expression was shown to be elevated in mice treated with bleomycin and bronchiolar and alveolar epithelial cells, as well as myeloid cells showing increased caspase-1 immunoreactivity in both nucleus and cytoplasm [83]. A decade later, it was suggested that inflammasome signaling in airway epithelial cells may play an important role in the pathogenesis of diseases like COPD, as compounds such as LPS and CpG were found to induce the releases of IL-1*β* from human bronchial epithelial cells [84]. Next to these environmental triggers, mechanical stretch was shown that same year to induce enhanced IL-1*β* levels in the supernatants of alveolar epithelial cells [85]. In recent years the panel of mediators able to activate the inflammasome-dependent caspase-1 activity and IL-1*β* release from (primary) lung epithelial cells has expanded rapidly to include* Pseudomonas aeruginosa*, simvastatin [86],* influenza A* [19, 87, 88], RSV [89], and* Rhinovirus* [90]. The list of activators seems to be unlimited. The membrane attack complex of complement, apart from its classical role of lysing cells, can also trigger a range of nonlethal effects on cells, including driving inflammation. Recent findings demonstrated that sublytic attack by the membrane attack complex of complement leads to caspase-1 activation as well as IL-1*β* secretion in primary human lung epithelial cells [91]. Another class of inflammasome activators in lung epithelial cells includes noxious inhaled particles. A panel of inflammasome-dependent mediators was shown to be released by bronchial epithelial cells following crystalline silica exposure [7] and Tran et al. demonstrated induction of IL-1*β* and NLRP3 protein by the proinflammatory stimulus LPS and the combination of IFN-*γ* with LPS in primary cell cultures of NHBE cells [92]. Hirota and his colleagues characterized airway epithelial NLRP3 inflammasome-mediated immune responses to urban particulate matter exposure and found significant increases in airway epithelial NLRP3 inflammasome-mediated production of IL-1*β in vitro*, results that were corroborated* in vivo* [93].

For interleukin-18, a prolific cytokine involved in many immune responses already issued, literature research revealed that its immunoreactivity in airway epithelial cells was first investigated during early stages of host defense within the bronchial epithelium of biopsies obtained from control subjects and patients with sarcoidosis or asthma [94]. Later, Western blot analysis showed that the 18.3 kDa mature form of IL-18 appeared in whole cell lysate of* Mycobacterium tuberculosis*-stimulated alveolar type II cells, whereas both nonstimulated and* Mycobacterium tuberculosis*-stimulated alveolar type II cells contained abundant 24 kDa pro-IL-18. These results indicated that* Mycobacterium tuberculosis* upregulates IL-18 expression at both transcriptional and posttranscriptional levels [95], implying the involvement of caspase-1 enzymatic activity and therefore inflammasome activation. Piper et al. furthermore found that IL-18 was released from* Rhinovirus*-infected lung epithelia. The release was not associated with cell death but was dependent on caspase-1 catalytic activity [90].

The endogenous danger protein HMGB1 was shown to be released from A549 cells infected with virulent* Legionella pneumophila* in association with caspase-1 activity [96]. HMGB1 levels were furthermore found to be elevated in cell supernatant from rat alveolar type II cell monolayers that underwent scratch wounding [97]. Moreover, mechanical stretch significantly increased HMGB1 protein expression in A549 cells [85]. These results are important in the context of injury, since epithelial crosstalk to neighboring cells is important for normal as well aberrant repair, such as in the case of fibrosis. HMGB1 was included in the panel of inflammasome-dependent mediators that were released following crystalline silica exposure of bronchial epithelial cells [7].

Epithelial cells express and secrete not only cytokines and alarmins upon exposure to endogenous or exogenous inflammasome activators but also bFGF. The FGFs are involved in morphogenesis, wound repair, inflammation, angiogenesis, and tumour growth and invasion and require the glycosaminoglycan (GAG) side chains of heparin sulphate proteoglycans for high affinity binding to their specific receptors [98]. Late 20th century, bronchial epithelial cells were shown to secrete bFGF which positively impacted myofibroblast proliferation in an animal model of asthma. A role for epithelial cells in the expression and release of bFGF from heparan sulphate binding sites in bronchial asthma was defined a couple of years later [99]. Treatment of human fibroblasts with caspase-1 inhibitors significantly reduced the amount of secreted bFGF [4]. The lung epithelium is a major source of bFGF as shown by* Rhinovirus*-induced bFGF release in a model that mimics features of airway remodeling [72]. We furthermore showed that crystalline silica exposure of bronchial epithelial cells caused bFGF release [6] which was inflammasome- and particle uptake-dependent [7]. A pivotal role of surface reactivity of crystalline silica to inflammasome activation was recently demonstrated in cultures of epithelial cells with evidence of the inhibitory capacity of the antioxidant TRX to inflammasome activation [100]. Importantly, our studies showed that the panel of silica-induced NLRP3 inflammasome-dependent mediators released from airway epithelium leads to fibroblast proliferation, a characteristic of multiple lung diseases. These findings are paralleled by work of Hussain et al., in which it is evidenced that multiwalled carbon nanotubes induce a NLRP3 inflammasome-dependent, but TGF-*β* independent, profibrotic response in human bronchial epithelial cells [101].

In contrast, there are publications that demonstrate that lung epithelial cells are not able to secrete IL-1*β* upon exposure to different microorganisms and particulates among others [102, 103]. This section however summarizes a vast body of evidence that lung epithelium participates in early first-line immune defenses via activation of the inflammasome. The expression of IL-18, IL-1*β*, and bFGF as well as HMGB1 by these cells demonstrates its participation in the initial response to encounters with foreign molecules. The described mediators may play a prominent role in the cascade of subsequent steps of the immune response in an autocrine and paracrine as well as chemotactic manner.

**Figure 4:**
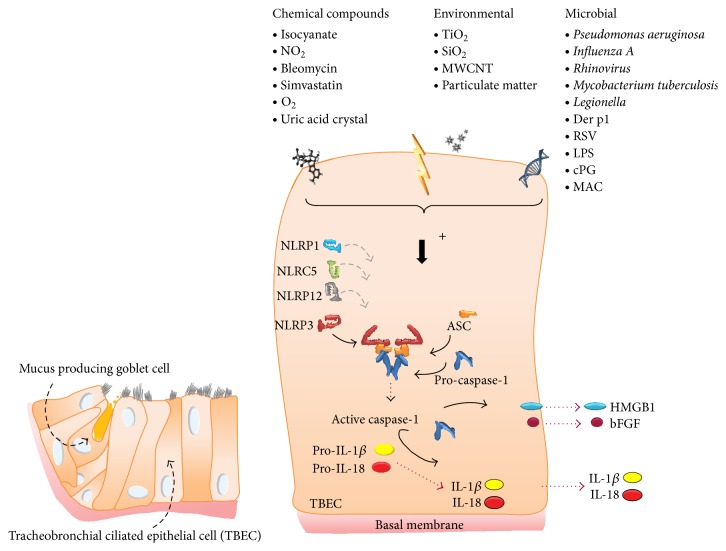
Schematic representation of pseudostratified columnar epithelium of the lung indicating a variety of environmental and microbial molecules that is able to activate the inflammasome with a subsequent release of cytokines, alarmins, and growth factors.

To date, only a subset of inflammasomes has been described in lung epithelial cells so far (Figure 4). NLRP1 was reported to contribute to the immune response in lung epithelial cells and alveolar macrophages [25]. NLRP3 inflammasome presence and activation in lung epithelial cells was demonstrated as well [92, 93]. Of most caspase-1 activating inflammasomes that have been studied well, NLRP12 is a unique NLR that has been shown to attenuate inflammatory pathways in biochemical assays and mediates the lymph node homing of activated skin dendritic cells in contact hypersensitivity responses. Although its expression was shown in lung cells, the overall development of allergic airway disease and airway function was not significantly altered by overall NLRP12 deficiency. This suggests that NLRP12 does not play a vital role in regulating airway inflammation in this model [104]. Upon* Rhinovirus* pathogenesis, the contribution of NLRP3 and NLRC5 inflammasomes and IL-1*β* secretion in* Rhinovirus* pathogenesis was investigated and revealed that both inflammasomes act in a cooperative manner during the assembly by sensing intracellular Ca^2+^ fluxes and triggering IL-1*β* secretion in primary human bronchial epithelial cells [105]. Additionally, the importance of inflammasome signaling in animal models representing a cadre of lung diseases such as asthma, COPD, and acute lung injury as well as fibrosis and pneumoconiosis among others has been shown by many groups [106–114] and was recently reviewed by Brusselle et al. [115]. However no focus on the epithelium is present in any model.

This section evidences that the surface epithelium of the conducting airways can be considered a constitutive primary participant in innate immunity with strong evidence that epithelial dysfunction is involved in the development of inflammatory disorders of the lung and could be a plausible target for therapeutic interventions. Often though, as indicated in the preceding paragraph the importance of inflammasome activation in the epithelium in animal models is not primarily approached. Therefore conditional knockout models or epithelial-specific transgenic animal studies will be a necessity.

## 6. **Urogenital Epithelium**

**Figure 5:**
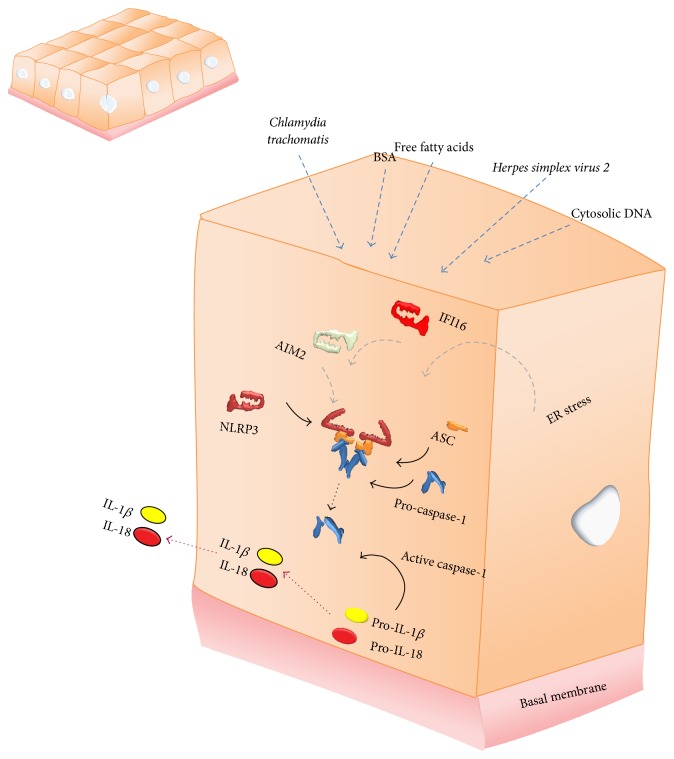
Schematic representations of simple cuboidal epithelial cells lining the urogenital tract in which different inflammasomes have been described to be activated by independent instigators triggering the release of inflammasome readouts.

With respect to cells lining the urogenital tract, evidence suggests that inflammasomes, next to other PPRs, have important roles in associated diseases through regulation of inflammatory and tissue-repair responses to infection and injury [116]. First, on the subject of human kidney diseases such as Wegener's granulomatosis and in experimental models of glomerulonephritis, glomerular as well as tubular epithelial cells have been shown to synthesize and release IL-1*β*, constitutively [117–119]. In a recent study that analyzed the processing of caspase-1, IL-1*β*, and IL-18 after unilateral ureteral obstruction (UUO) in mice reflecting chronic kidney disease, it was shown that NLRP3 has a biological function in both hematopoietic and renal epithelial compartments during renal injury. Additionally, in models of ischemic tubular necrosis and obstruction-induced epithelial-mesenchymal transition, an important role for caspase-1 and IL-18 has been demonstrated under hypoxic conditions and in the absence of vascular effects [120–122]. Other cells lining epithelial tracts in contact with the environment conveying inflammasomes are prostate epithelial cells expressing AIM2 with increased caspase-1 activity in an experimental model of benign prostate hyperplasia (BPH), and human cervical epithelial cells expressing AIM2 and IFI16 inflammasomes following* Chlamydia trachomatis* and* herpes simplex virus 2*, respectively [123–125]. Although the amount of literature on inflammasome activation in these organs is relatively scarce, other studies demonstrate a pivotal role of the presence and activation of various inflammasomes in the epithelium of urogenital organs exposed to the environment [126–128] (Figure 5).

## 7. Conclusion

Epithelial cells form an interface between the body and the environment. Therefore, they are important guardians for the detection of danger signals and the consecutive initiation of an inflammatory response. As presented in this review, each organ and cell type express different sensor subtypes with discrepancy in the release of various mediators. It should be emphasized that it is very possible that manifold inflammasomes are important in multiple epithelial cell types and become activated to either overcome detrimental signaling or to cooperate in a constructive fashion combating the disease. Likewise, with respect to relatively lower concentrations of cytokines released from epithelial cells versus the myeloid compartment, it could be considered that first-line barrier epithelial cells, in contact with many potential danger signals, preferably should not produce high amounts of these very potent inflammatory cytokines and alarmins as it would be harmful for the microenvironment to have a constant “high-alarm situation.” Additionally, many more epithelial cells are present in these organs as opposed to, for instance, macrophages; therefore activated epithelial cells may relay equally large and biologically significant immune signals that build up the important contribution in global inflammasome activation at organ level.

This review demonstrates that inflammasome activation and subsequent secretion of “alarming” proteins is not restricted to macrophages, indicating that epithelial cells should be considered as highly important cells in innate immune signaling. In future research, epithelial-specific conditional knockout models and transgenic animal studies will be a necessary approach to determine this important contribution more profoundly.

## Abbreviations

AEC: Alveolar epithelial cell
AIM2: Absent in melanoma 2
ALR: AIM2-like receptor
ASC: Apoptosis-associated speck-like protein containing a CARD
ATP: Adenosine-5′-triphosphate
bFGF: Basic fibroblast growth factor
CARD: Caspase recruitment domain
DAMPs: Danger-associated molecular patterns
*Der p1*: * Dermatophagoides pteronyssinus 1*
DNA: Deoxyribonucleic acid
dsDNA: Double-stranded deoxyribonucleic acid
DSS: Dextran sodium sulphate
ELISA: Enzyme-linked immunosorbent assay
HIN-200: 200 amino acid hemopoietic IFN-inducible nuclear proteins
HMGB1: High-mobility group box 1
HPV: * Human papillomavirus*
IBD: Irritable bowel disease
ICE: Interleukin-1 converting enzyme
IEC: Intestinal epithelial cell
IFI16: Interferon alpha-inducible protein 16
IFIX: Interferon inducible protein X
IFN: Interferon
IL-18: Interleukin-18
IL-1R: Interleukin-1 receptor
IL-1*α*: Interleukin-1*α*
IL-1*β*: Interleukin-1*β*
LPS: Lipopolysaccharide
LRR: Leucine-rich repeat domain
MAC: Membrane attack complex
MNDA: Myeloid nuclear differentiation antigen
MSU: Monosodium urate
MTB: * Mycobacterium tuberculosis*
MWCNT: Multiwalled carbon nanotubes
MyD88: Myeloid differentiation primary response protein
NACHT: Nucleotide-binding and oligomerization domain
NADPH: Nicotinamide adenine dinucleotide phosphate-oxidase
NAIP: NLR family, apoptosis inhibitory protein
NK cells: Natural killer cells
NLR: Nucleotide-binding domain leucine-rich repeat containing receptors
NLRC4: Nucleotide-binding domain leucine-rich repeat containing receptors with a CARD domain 4
NLRC5: Nucleotide-binding domain leucine-rich repeat containing receptors with a CARD domain 5
NLRP1: Nucleotide-binding domain leucine-rich repeat containing receptors with a pyrin domain 1
NLRP3: Nucleotide-binding domain leucine-rich repeat containing receptors with a pyrin domain 3
NLRP6: Nucleotide-binding domain leucine-rich repeat containing receptors with a pyrin domain 6
NLRP7: Nucleotide-binding domain leucine-rich repeat containing receptors with a pyrin domain 7
NLRP10: Nucleotide-binding domain leucine-rich repeat containing receptors with a pyrin domain 10
NLRP12: Nucleotide-binding domain leucine-rich repeat containing receptors with a pyrin domain 12
NOD: Nucleotide-binding oligomerization containing domain
P2X7: Purinergic receptor P2X, ligand-gated ion channel, 7
PAMPs: Pathogen-associated molecular patterns
PRR: Pattern recognition receptor
PYD: Pyrin domain
PYHIN: Pyrin and HIN200 domain-containing protein
RAGE: Receptor for advanced glycation end-product
RNA: Ribonucleic acid
ROS: Reactive oxygen species
RSV: * Respiratory syncytial virus*
SiO_2_: Silicon dioxide
siRNA: Small interfering RNA
TGF-*β*: Transforming growth factor *β*
TiO_2_: Titanium dioxide
TNBSA: 2,4,6-Trinitrobenzene sulfonic acid
TNF-*α*: Tumor necrosis factor-*α*
WCL: Whole cell lysate.

## Gloss

The immune response of the body is determined by a complex interplay between danger molecules and the host, in which the recognition of these molecules by myeloid cells has been linked with cytosolic sensors such as the inflammasome. Over the past decade, there has been tremendous progress in our knowledge of the central roles of NOD-like receptor (NLR) and non-NLR inflammasomes in the immune responses in many cell types. Here we provide evidence that epithelial cells lining multiple barriers between the inner body and its possible invaders are equipped with functional inflammasomes.
